# Development of a protocol for detection of SARS-CoV-2 in sputum and endotracheal aspirates using Cepheid Xpert Xpress SARS-CoV-2

**DOI:** 10.1099/acmi.0.000176

**Published:** 2020-11-12

**Authors:** Michael Malczynski, Saba Rezaeian, Javier Rios, Laura Dirnberger, Wanda Polanco, Teresa Zembower, Chao Qi

**Affiliations:** ^1^​ Clinical Microbiology Laboratory, Department of Pathology, Northwestern Memorial Hospital, Chicago, IL, USA; ^2^​ Division of Infectious Diseases, Department of Medicine, Northwestern University Feinberg School of Medicine, Chicago, IL, USA; ^3^​ Department of Pathology, Northwestern University Feinberg School of Medicine, Chicago, IL, USA

**Keywords:** Cepheid Xpert Xpress, endotracheal aspirate, rapid detection, SARS-CoV-2, sputum

## Abstract

Sputum and endotracheal aspirates (ETs) are not among the vendor-approved specimens for the Cepheid Xpert SARS-CoV-2 assay. However, they are the common lower respiratory tract specimens submitted for laboratory diagnosis. Testing for severe acute respiratory syndrome coronavirus 2 (SARS-CoV-2) in lower respiratory tract samples is required for the discharge of patients from coronavirus disease (COVID) units at some institutions. We developed a protocol used for testing unliquified viscous sputum or tracheal aspirate with the Cepheid Xpert SARS-CoV-2 assay.

## Introduction

Hospitals treating coronavirus disease 2019 (COVID-19) patients are facing the challenge of discharging recovered patients from COVID-positive units to COVID-negative units or to long-term care facilities. In a setting where the hospital has a limited number of beds on a COVID unit, there is a need for early discharge after treatment and recovery. Concerns for early discharge are that prolonged viral shedding in sputum and endotracheal aspirates from recovered patients can cause local transmissions. A study of paired nasopharyngeal (NP) swab and sputum samples taken on the same patient admission showed that the shedding of live viruses occurred for a longer period and at a higher level in sputum compared to NP swabs [1]. In addition, studies have demonstrated that viruses can be detected in asymptomatic and mildly symptomatic case patients for more than 2 weeks from lower respiratory tract specimens [2]. Two negative RT-PCR results, at least one from a lower respiratory tract sample, from consecutive days, was one of the criteria for discharging patients from a COVID unit at our institution. Sputum and endotracheal aspirates (ETs) are the common lower respiratory tract specimens submitted for testing. These specimen types are not among the vendor-approved specimens for the Cepheid Xpert SARS-CoV-2 assay. Direct testing of sputum or endotracheal aspirate samples with the Xpert SARS-CoV-2 assay commonly produced invalid results when using the vendor-recommended protocol. Here, we report the development of a protocol used for testing unliquified viscous sputum or tracheal aspirate with the Cepheid Xpert SARS-CoV-2 assay.

## Methods

A total of 50 samples from hospitalized patients, including 23 ET and 27 sputum samples, were collected and tested retrospectively. The majority of these samples were from patients in a COVID unit. A few samples collected from patients on non-COVID units were included to serve as negative controls. Preparing samples for testing included the following steps: (1) a sterile swab (non-cotton tipped) was placed into the tracheal aspirate or sputum specimen; (2) the swab tip was mixed thoroughly in the specimen; (3) the swab tip was broken into 500 μl 0.9 % sterile saline; (4) the tube was vortexed for 10 s; (5) 300 μl of suspension was transferred into the Xpert Xpress SARS-CoV-2 cartridge using the transfer pipette provided in the Cepheid Assay kit. The test was then run according to the manufacturer’s instructions [3].

The diagnostic performance of the sputum/ET testing procedure was assessed by comparison to NP testing with the Xpert Xpress SARS-CoV-2 assay. A positive test result was considered to be a true positive when the sample was from a confirmed COVID patient with a previous positive NP test result generated from the Abbott ID NOW, Cepheid Xpert SARS CoV-2 Assay, or the CDC COVID-19 Assay in the past 30 days. A negative test result was considered to be a true negative when the sample was from a patient on a non-COVID unit. When a sputum or an ET sample from a patient on a COVID unit was deemed SARS CoV-2-negative, the result was considered to be a true negative when one or more NP or BAL samples from the same patient produced a COVID-negative result within one day prior to or after the sputum/ET testing. A 2×2 table was constructed to compare sputum/ET and NP test results and the percentage agreement of the two methods was calculated [4].

## Results

Among the 50 samples tested, 26 were positive and 24 were negative. No invalid PCR results were produced. No discrepant result was identified when compared to the NPs and BALs, which resulted in a positive, negative and overall percentage agreement of 100 % (Table 1). The average cycle threshold values for targets E and N2 were 22.2±11.4 and 30.4±7.5, respectively.

**Table 1.** Contingency table showing the diagnostic performance of the Xpert Xpress SARS-CoV-2 assay for the detection of SARS-CoV-2 in sputum and NP samples

		NP-positive	NP-negative
Sputum/ET	Positive	26	0
	Negative	0	24

Positive percentage agreement, 100 %.

Negative percentage agreement, 100 %.

Overall percentage agreement, 100 %.

NP, nasopharyngeal; ET, endotracheal aspirate

There were five patients who had NP and sputum/ET samples on the same day. A comparison of the average cycle threshold values of test results for NP and sputum/ET specimens showed no significant difference (Table 2).

**Table 2.** Average cycle threshold values for targets E and N2 in nasopharyngeal and sputum specimens (*n*=5) using the Xpert Xpress SARS-CoV-2 assay

	E	*C*_t_ range	N2	*C*_t_ range
Nasopharyngeal	28.5±5.0	21.3–35.1	30.7±5.3	23.3–37.8
Sputum/ET	29.1±7.4	16.6–37.6	31.5±7.5	19–40.5

E, envelope gene; Ct, cycle threshold value; N2, nucleocapside gene; ET, endotracheal aspirate

The US Food and Drug Administration (FDA) has issued an emergency authorization for use of the Cepheid Xpert Xpress SARS-CoV-2 for rapid detection of SARS-CoV-2 using specimens from a nasopharyngeal swab or nasal wash/aspirate. Sputum and ETs are not among the approved specimens. We developed a protocol for the detection of SARS-CoV-2 in sputum and ET samples with the Xpert Xpress SARS-CoV-2 Assay. The protocol was simple and did not require pretreatment of the specimens. No invalid results were produced when using the protocol and the detection of SARS-CoV-2 in sputum/ETs was in 100 % agreement with testing of NP and BAL specimens.
